# Malignant granular cell tumor in the thoracic wall: A case report

**DOI:** 10.3389/fonc.2022.895924

**Published:** 2022-09-20

**Authors:** Shengnan Gao, Bin Xing, Sun Lichao, Jie Luo, Jiao Tang, Ye Wang, Xiaoyan Zhang

**Affiliations:** ^1^ Department of Clinical Laboratory, Cancer Hospital, Chinese Academy of Medical Sciences and Peking Union Medical College, National Cancer Center/National Clinical Research Center for Cancer/State Key Laboratory of Molecular Oncology, Beijing, China; ^2^ Department of Pulmonary and Critical Care Medicine, Center of Respiratory Medicine, China-Japan Friendship Hospital, Beijing, China; ^3^ National Center for Respiratory Medicine, Beijing, China; ^4^ Institute of Respiratory Medicine, Chinese Academy of Medical Sciences, Beijing, China; ^5^ National Clinical Research Center for Respiratory Diseases, Beijing, China; ^6^ Emergency department, China Japan Friendship Hospital, Beijing, China; ^7^ Department of Pathology, China-Japan Friendship Hospital, Beijing, China; ^8^ Department of internal medicine, China Nuclear Industry Beijing 401 Hospital, Beijing, China; ^9^ Beijing University of Chinese Medicine, Beijing, China

**Keywords:** granulosa cell tumor, thoracic wall, chest wall, Schwann cells, neuroectodermal, soft tissue tumor, MPNST

## Abstract

Granulosa cell tumor (GCT) is a rare tumor that originates from neural/Schwann cells. GCTs can occur at any age and at any site in the body. The most common site is the tongue, followed by the mammary gland, upper respiratory tract ( throat and bronchus), and gastrointestinal tract (esophagus, large intestine and perianal area, stomach, small intestine, and bile duct). Malignant GCTs account for less than 1%–2% of all GCTs. Fewer than five GCTs in the thoracic wall have been reported, almost all of these benign. Here, we report a new case of malignant GCT of the thoracic wall, with rib invasion and pleural metastasis, in an Asian male. Microscopic examination revealed round, granular cells with eosinophilic cytoplasm and without prominent atypia. Despite these findings the disease showed rapid clinical progression. In summary, the tumor, although histologically ‘benign’, was clinically ‘malignant’.

## Introduction

Granular cell tumor (GCT) is a rare tumor that originates from neural or neuroectodermal cells, and was first described in 1945 by Ravich et al. (1) Malignant GCTs account for less than 1%–2% of GCTs (2), which can occur in the lower extremities (3), breast (4), thyroid (5), abdominal wall (6), and bronchus (7). GCTs rarely develop in the thoracic wall. Malignant GCT is characterized by invasion and metastasis, which represent a poor prognosis Invasion and metastasis are characteristics of malignant variation, and represent a poor prognosis. To our knowledge, fewer than five cases of GCT in the chest wall have been reported, and almost all of these were benign. Here, we report a malignant GCT that occurred in the thoracic wall, and summarize the clinical profile, histopathological features, and diagnosis of this tumor.

## Patient information

A 76-year-old Asian man with a 1-year history of burning pain in the right shoulder and right armpit worsened after fatigue. A palpable mass had been detected in in the patient’s right anterior chest wall 3 months previously and had gradually enlarged. On physical examination, a non-tender firm mass was palpated in the right anterior chest wall. The skin around the mass was intact, and no erythema was noted.

## Clinical findings

**Figure 1:**
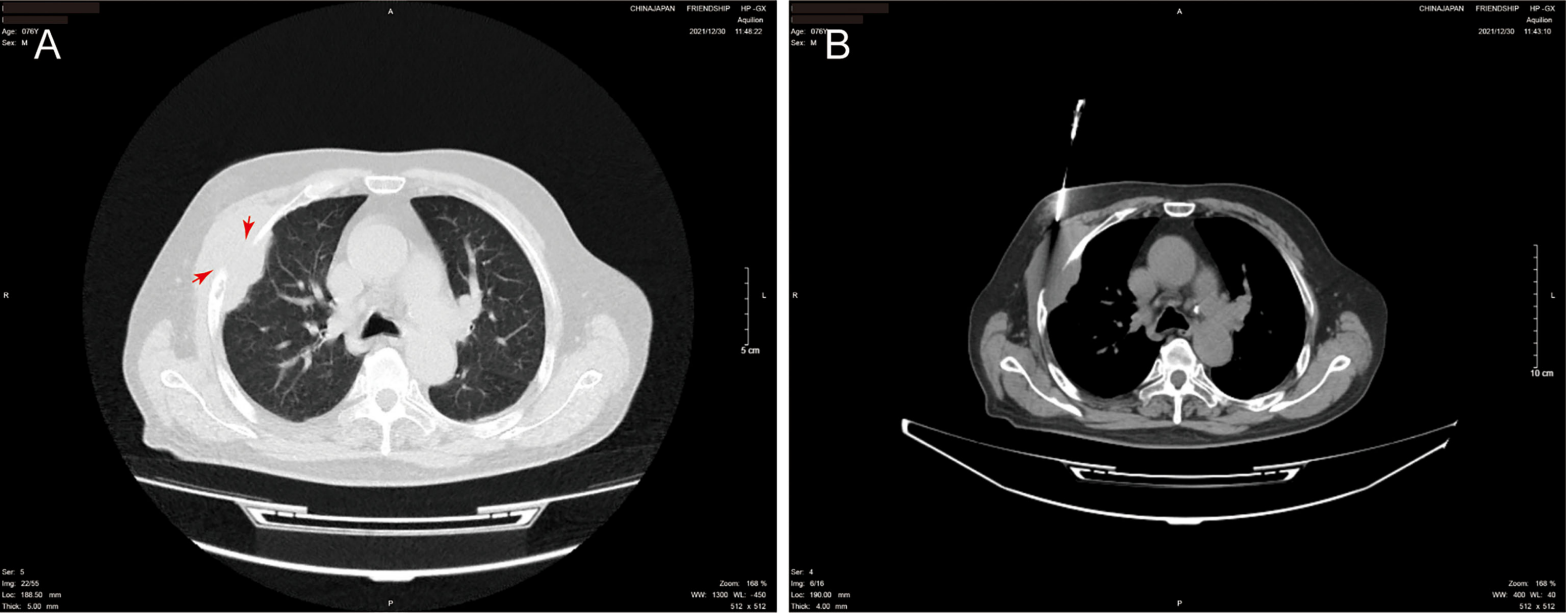
Chest computed tomography (CT) scans. **(A)** Pulmonary window showing the maximum cross-section of the mass (89 × 42 mm). The image shows the third and fourth ribs, which exhibit insect-like bone destruction; the adjacent pleura is thickened (red arrow). **(B)** Mediastinal window obtained under CT-guided core needle biopsy.

A computed tomography (CT) scan of the chest showed the shadow of a soft tissue mass (89 × 42 mm) on the right chest wall. The focus surrounded the third and fourth ribs, and showed insect-like bone destruction and thickened adjacent pleura (**Figure 1A**).

**Figure 2:**
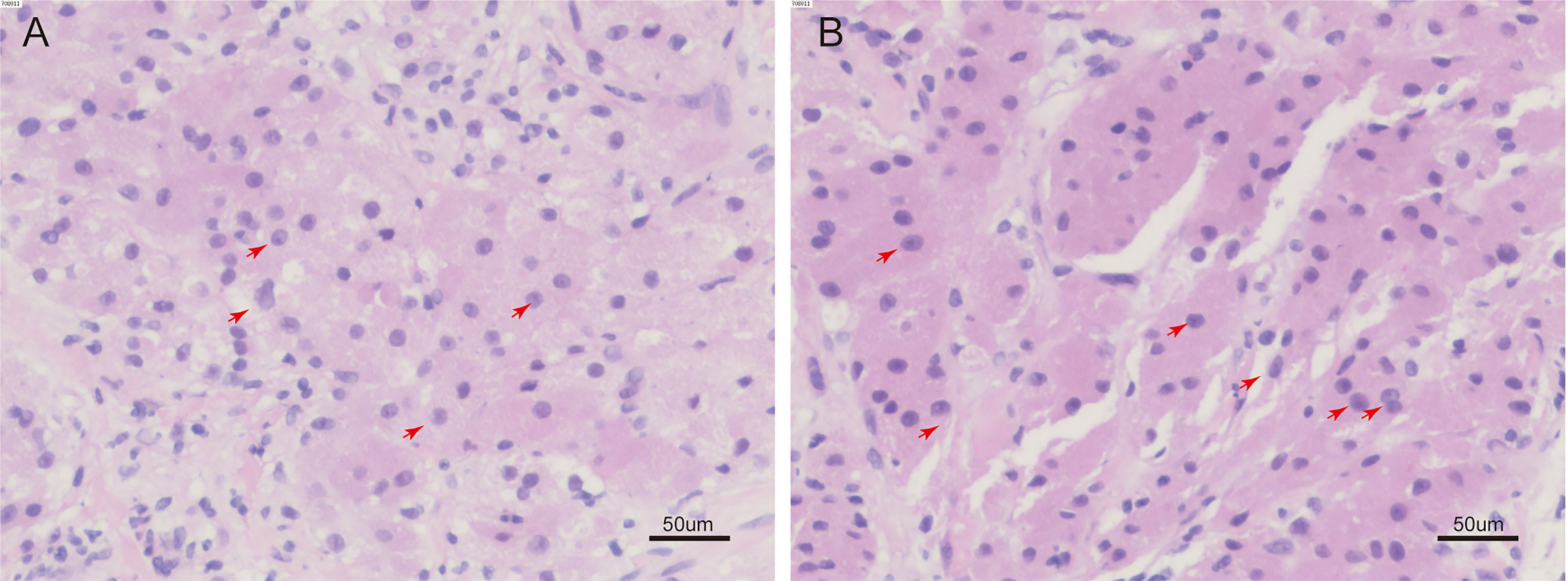
H&E staining of tumor tissue. **(A, B)**, some cells are spindle shaped, the nucleus of the tumor cell can be seen to be slightly enlarged, with obvious nucleoli, a high nucleus-to-cytoplasmic ratio, and nuclear fission. (red arrow) (H&E, 200 ×).

**Figure 3:**
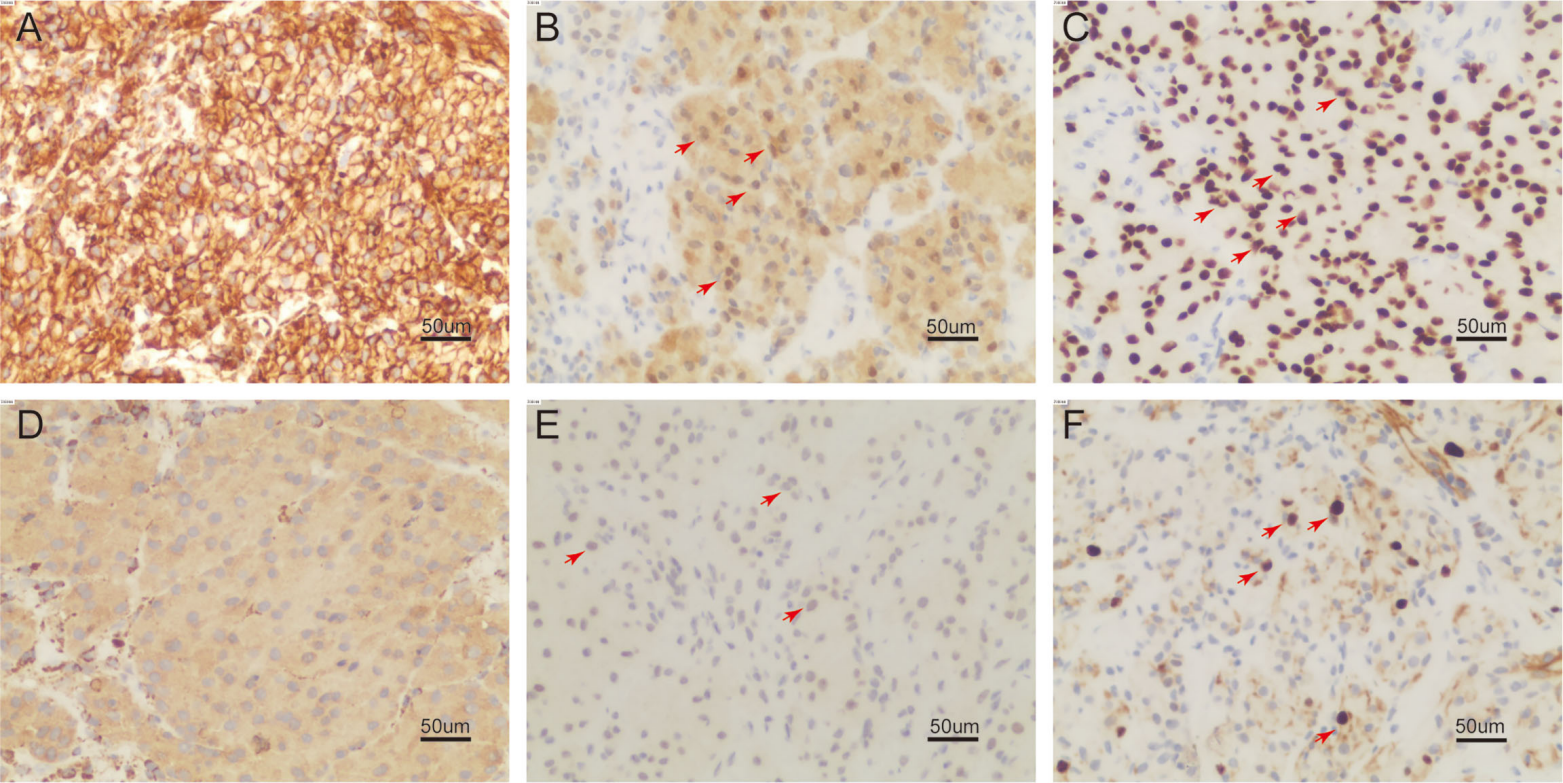
On immunohistochemistry examination (200 ×), the tumor cell is reactive to vimentin **(A)**, S-100 **(B)**, SOX-10 **(C)**, KP-1 **(D)**, PHH3 showed positive mitotic cells **(E)**, Ki-67 reacted more than 8% **(F)** (positive region in **(B, C, E, F)** marked with red arrow).

On 30 December 2022, we carried out CT-guided core needle biopsy of the mass and obtained two samples of grayish-white tissue (**Figure 1B**). Microscopic examination revealed many round, granular cells with eosinophilic cytoplasm and without prominent atypia. The nuclei of the tumor cells were slightly enlarged, and some nuclei contained conspicuous nucleoli. Some cells had a high nucleus-to-cytoplasm ratio, and cells were spindle-shaped occasionally. Nuclear fission was seen (**Figure 2**). Immunohistochemistry showed that these cells were negative for cytokeratin AE1/AE3, cytokeratin 5/6/7, TTF-1, napsin A, P40, CgA, Syn, D2-40, CD38, HMB45, desmin, α-SMA, MyoD1, SF-1, and Melan-A, but were positive for S-100, vimentin, calretinin, KP-1, SOX-10, and inhibin-α. Ki-67 reacted with more than 8% of cells. Phosphorylated Histone H3 (PHH3) showed positive mitotic cells (**Figure 3**). In addition, periodic acid–Schiff staining was negative.

**Figure 4:**
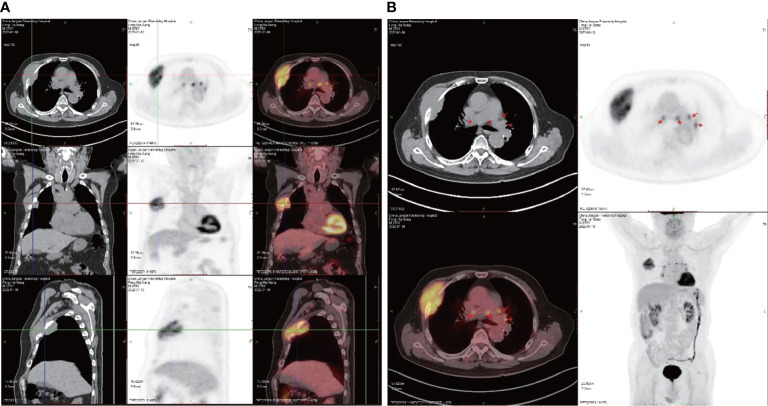
Positron emission tomography (PET) CT scans. **(A)** The tumor in frontal, lateral, and transverse section. **(B)** Multiple lymph nodes showing fluorodeoxyglucose uptake in the mediastinum and hilum (SUVmax 7.4).

Positron emission tomography (PET) CT (**Figure 4**) revealed a soft tissue mass on the right chest wall between the third and fourth ribs, with a cross-section of approximately 86 × 36 mm, destruction of the third and fourth ribs, and increased fluorodeoxyglucose (18F-FDG) uptake [maximum standardized uptake value (SUVmax) 8.2] (**Figure 4A**). Uptake of fluorodeoxyglucose by multiple lymph nodes in the mediastinum and hilum (SUVmax 7.4) was observed (**Figure 4B**). There were many small nodules adjacent to the pleura and interlobar pleura: the largest ones were approximately 4 mm in diameter. In addition, no abnormal fluorodeoxyglucose uptake was detected in either of the lungs or in the left pleura. A diagnosis of malignant GCT was made for this case.

We also conducted molecular pathology tests to detect treatment-related gene mutations. No mutation was detected in exons 18/19/20/21 of epidermal growth factor receptor(EGFR), exon 2 of K-rat sarcoma viral oncogene (KRAS), exon 3 of N-rat sarcoma viral oncogene (NRAS), 600 of exon 15 (V600E) of v-raf murine sarcoma viral oncogene homolog B1(BRAF), exon 14 of mesenchymal-epithelial transition factor (MET), the total length of phosphatidylinositol-4,5-bisphosphate 3-kinase catalytic subunit alpha (PIK3CA) and human epidermal growth factor receptor 2 (HER2). There was no gene fusion of anaplastic lymphoma kinase (ALK), proto-oncogene tyrosine-protein kinase (ROS1), and proto-oncogene tyrosine-protein kinase recepto r(RET).

All procedures carried out in studies involving human participants followed the ethics standards of the national research committee(s). The patient’s written informed consent was obtained.

## Discussion

Since GCTs were first described by Abrikosoff in 1926 (22), several cell lineages have been proposed as the origin, including monocytes/macrophages, neuroendocrine cells, fibroblasts, myoblasts/myofibroblasts, Schwann cells, and undifferentiated mesenchymal cells. Although the origin of GCTs is still controversial, a large amount of morphological, immunohistochemical, and ultrastructural evidence supports a neural/Schwann cell origin. Therefore, GCTs are currently recognized as a peripheral nerve tumor known as granulosa cell schwannoma and granulosa cell neurofibroma.

Granulosa cell tumors can occur at any age, but are mostly found in adults aged 40–70 years, and are seen slightly more often in women. GCTs are widely distributed in the skin and subcutaneous tissue of the head and neck, trunk, and limbs. The most common site is the tongue, followed by the mammary gland, upper respiratory tract (throat and bronchus), and gastrointestinal tract (esophagus, large intestine and perianal, stomach, small intestine, and bile duct). GCTs usually present as a painless mass; however, malignant GCTs can cause persistent pain at the site of occurrence. Approximately 10%-20% cases presented with multiple tumors in various lesions, multiple tumors are present, but the number of lesions varies greatly (8, 9). Fewer than five GCTs have been reported in the chest wall, and all of these were benign. Malignant GCT of the thoracic wall, as identified in our case, has not previously been reported.

Granulosa cell tumors appear as ill-defined nodules, most of which are < 3 cm in diameter (malignant GCTs are often > 5 cm in diameter) and in section are light yellowish brown or grayish yellow. Most tumor cells are found in the dermis, subcutaneous layer, or the submucosa. The tumor cells are round, polygonal, or spindle shaped, with rich cytoplasm and fine eosinophilic granules. Thick eosinophilic globules with a halo, called ‘pustulo-ovoid bodies of Milian (POB)’, can often be seen (10). PAS staining is positive and resistant to amylase digestion. Composite lysosomes can be seen under electron microscope. The tumor cell nuclei vary greatly, from small and dense staining to a large vacuolated nucleus, and nuclear fission is rare. Focal cells may demonstrate slight to moderate atypia, with multinucleated giant cells. Malignant GCTs may have enlarged nuclei, obvious nucleoli, and increased mitotic and even pathological mitotic images. The arrangement of tumor cells is diverse, and cells can form nests, bands or, sometimes, cluster around a nerve. istolo may also infiltrate surrounding tissues (e.g., fat, and striated muscle). Neoplastic necrosis suggests malignancy.

Immunohistochemistry showed that the tumor cells expressed S-100, SOX-10, and neuron specific enolase (NSE), some expressed CD68 and α-inhibin. Smooth muscle actin (SMA), CD34, cytokeratin (CK), epithelial membrane antigen (EMA), melanoma marker (HMB45), and Melan-A were negative, with a low Ki-67 index. Consistent with our report, immunohistochemistry is positive for S-100, vimentin, calretinin, KP-1, SOX-10, inhibin, and PHH3. Ki-67 reacted with more than 8% of cells. Cytokeratin AE1/AE3, cytokeratin 5/6/7, thyroid transcription factor-1 (TTF-1), napsin A, P40, CgA, Syn, D2-40, CD38, HMB45, desmin, α-SMA, Myogenic Differentiation 1 (MyoD1), ssplicing factor 1 (SF-1), and Melan-A were negative.

Considering the location of the neoplasm in the case we reported, the principal differential diagnosis to be considered is malignant pleural mesothelioma. First, the tumor in our case has a unique GCT arrangement and morphology (round cells withrich cytoplasm and fine eosinophilic granules). Second, due to their neurogenic origin, GCTs have different molecular markers from malignant pleural mesothelioma; for example, the former is marked by S-100 and SOX-10 and the latter by desmin and Ki-67. The high expression of S100 and SOX-10 in our case also supports the neurogenesis of the tumor. Third, in terms of clinical behavior, malignant pleural mesothelioma tends to cause pleural effusion but rarely involves chest wall tissue. A chest wall involvement in our case suggests a GCT diagnosis.

Malignant peripheral nerve sheath tumors (MPNSTs), given their Schwannian origin, should also be included in the differential diagnosis. Morphologically, MPNSTs are characterized by highly cellular fascicles of spindle cells, with hyperchromatic, tapering, and pleomorphic nuclei (11, 12). These histopathological features are inconsistent with the findings in our case, in which the tumor displayed round cells with granular and eosinophilic cytoplasm. In addition, MPNSTs usually express limited Schwann cell markers (S100 protein and SOX10), sometimes with focal or patchy staining, in contrast to the firm and uniform staining in this case (11, 13). Therefore, based on the pathological and molecular aspects of this case, we are more inclined to diagnose a GCT.

Finally, although malignant GCTs of the thoracic wall are rare, it is also important to consider the possibility of a malignant, rather than a benign, GCT, based on the tumor’s biological conduct and clinical characteristics. Malignant GCTs are rare, accounting for less than 2% of all GCTs. Malignant GCT was first reported by Ravich et al. in 1945. It is associated with a poor prognosis, manifested by a local recurrence rate of 32%–70%, a metastasis rate of 50%–62%, and a mortality rate of 39%–65% (2, 14–16). The criteria used to discriminate between benign and malignant GCTs have always been controversial given that the clinical course and pathology of GCTs are not completely consistent. Many GCTs initially thought to be benign result in subsequent relapse or metastasis and turn out to be malignant. Benchekroun reviewed that 2%-3% of reported cases were histologically benign’, but the clinical features presented as malignant. Many investigators consider ‘metastasis’ as the defining criterion of malignant GCTs (17–19). Fanburg-Smith et al. (2) found no ‘benign’ morphology in their study of 46 cases of malignant GCT. The authors described six histological features characteristic of malignant GCT: (1) an increased nucleus-to-cytoplasm ratio, (2) nuclear pleomorphism, (3) tumor necrosis, (4) spindle cell degeneration, (5) vacuolar nucleus and a large nucleolus, and (6) more than two mitotic figures 10 per high-power fields (HPFs). They considered a GCT to be ‘atypical’ if it displayed one or two of these features, and ‘malignant’ if it displayed three to six Features. Subsequent investigators have proposed other diagnostic criteria. For example, Nasser ‘et al. (19) described a diagnostic system according to which GCTs are considered benign or of uncertain malignant potential according to whether there is ‘necrosis’ and/or ‘more than two mitotic figures per 10 HPFs’. Machado et al. (17) classified tumors with malignant histological features as ‘GCT with increased risk of metastasis’, while tumors without malignant features were classified as ‘GCT with almost no metastatic potential’. Both groups believe that metastasis is the unique diagnostic criterion of malignant GCT.

In our opinion, these differences can be resolved by using Gamboa’s classification. As early as 1955, Gamboa proposed two types of malignant GCT (20). One, classified as Gamboa I, is clinically malignant but has a ‘benign’ istologically, ““and the other, classified as Gamboa II, is both clinically and histologically malignant. In practice, the Gamboa classification and the Fanburg-Smith criteria can be combined. If the tumor is confirmed to have metastasized, it should be diagnosed as malignant GCT, even if its morphology is ‘benign’. Otherwise, Fanburg-Smith criteria should be applied, and the tumor should be classified as benign/atypical/malignant GCT.

The histomorphology in this case showed benign features. However, the clinical behavior was more like that of a malignant GCT: not only did the mass proliferate rapidly, reaching a maximum diameter of 89 cm within 3 months of the initial presentation, but CT showed that the tumor tissue had invaded the ribs and revealed the presence of pleural metastases. The above histological and clinical features leads to the classification of the tumor as Gamboa I, and, therefore, a diagnosis of malignant GCT.

## Treatment

Surgical resection is the primary approach to the treatment of GCTs, in order to prolong survival, and widespread lymph node dissection is required in the case of malignant GCTs. Radiotherapy and chemotherapy are ineffective. Moten et al. (21) and Nasser et al. (19) believe that a large tumor size (larger than 5–10 cm) indicates a poor prognosis. The patient in this report refused surgical resection given their age, prognosis, and quality of life. To develop an individualized treatment plan, we carried out molecular pathology tests to detect mutations in treatment-related genes (exons 18/19/20/21 of *EGFR*, exon 2 of *KRAS*, exon 3 of *NRAS*, V600E of *BRAF*, exon 14 in *MET*, total length of *PIK3CA* and *HER2*, and gene fusion of *ALK*, *ROS1*, and *RET*). Unfortunately, no beneficial mutations were detected.

## Data availability statement

The original contributions presented in the study are included in the article/supplementary material. Further inquiries can be directed to the corresponding author.

## Ethics statement

Written informed consent was obtained from the individual(s) for the publication of any potentially identifiable images or data included in this article.

## Author contributions

SG composed the manuscript and literature review; JL and BX provided figures and pathology review; JT and YW collected clinical data; LS processed image analysis; XZ carried out the acquisition, comment, or interpretation of data for the work, revising it critically for important intellectual content, final approval of the version to be published, and agreement to be accountable for all aspects of the work in ensuring that questions related to the accuracy or the integrity of any part of the work is appropriately investigated and resolved.

## Conflict of interest

The authors declare that the research was conducted in the absence of any commercial or financial relationships that could be construed as a potential conflict of interest.

## Publisher’s note

All claims expressed in this article are solely those of the authors and do not necessarily represent those of their affiliated organizations, or those of the publisher, the editors and the reviewers. Any product that may be evaluated in this article, or claim that may be made by its manufacturer, is not guaranteed or endorsed by the publisher.
